# Effects of probiotic *Bacillus* as a substitute for antibiotics on antioxidant capacity and intestinal autophagy of piglets

**DOI:** 10.1186/s13568-017-0353-x

**Published:** 2017-02-28

**Authors:** Yang Wang, Yanping Wu, Baikui Wang, Xuefang Cao, Aikun Fu, Yali Li, Weifen Li

**Affiliations:** 1 Key Laboratory of Molecular Animal Nutrition of the Ministry of Education, Institute of Feed Science, College of Animal Sciences, Zhejiang University, Hangzhou, 310058 China; 2 Animal Nutrition and Human Health Laboratory, School of Life Sciences, Hunan Normal University, Changsha, 410006 China

**Keywords:** Piglets, Antibiotics, *Bacillus amyloliquefaciens*, Antioxidation, Autophagy

## Abstract

The objective of this study was to evaluate effects of probiotic *Bacillus amyloliquefaciens* (*Ba*) as a substitute for antibiotics on growth performance, antioxidant ability and intestinal autophagy of piglets. Ninety piglets were divided into three groups: G1 (containing 150 mg/Kg aureomycin in the diet); G2 (containing 75 mg/Kg aureomycin and 1 × 10^8^ cfu/Kg *Ba* in the diet); G3 (containing 2 × 10^8^ cfu/Kg *Ba* in the diet without any antibiotics). Each treatment had three replications of ten pigs per pen. Results showed that *Ba* replacement significantly increased the daily weight gain of piglets. Moreover, improved antioxidant status in serum and jejunum was noted in *Ba*-fed groups as compared with aureomycin group. Increased gene expression of antioxidant enzymes and elevated nuclear factor erythroid 2 related factor 2 (Nrf2) in jejunum was also observed in *Ba*-fed groups. Besides, *Ba* replacement significantly decreased jejunal c-Jun N-terminal kinase (JNK) phosphorylation compared with antibiotic group. Western blotting results also revealed that replacing all antibiotics with *Ba* initiated autophagy in the jejunum as evidenced by increased microtubule-associated protein 1 light chain 3 II (LC3-II) abundance. Taken together, these results indicate that replacing aureomycin with *Ba* can improve growth performance and antioxidant status of piglets via increasing antioxidant capacity and intestinal autophagy, suggesting a good potential for *Ba* as an alternative to antibiotics in feed.

**Electronic supplementary material:**

The online version of this article (doi:10.1186/s13568-017-0353-x) contains supplementary material, which is available to authorized users.

## Introduction

As growth promoters, antibiotics have enjoyed great popularity in animal husbandry in the past decades. However, with increasing public concerns regarding antibiotic-resistant pathogens, antibiotics have been forbidden in Europe since 2006 (Chu et al. 2013) and bans for antibiotic uses in feed are proposed in other many countries, including China, Korea, USA, etc. (Flynn 2011; Martin et al. 2015; Walsh and Wu 2016). Therefore, finding proper alternatives to antibiotics is important for the feed industry.

Probiotics are defined as “live microorganisms that, when administrated in adequate amounts, confer a health benefit on the host” (Araya 2002). Previous studies showed that probiotics have positive effects on pig health, including improving growth performance (Guerra et al. 2007; Giang et al. 2010), regulating immunity (Daudelin et al. 2011; Deng et al. 2013) and increasing survival rate of piglets (Sha et al. 2015). *Bacillus amyloliquefaciens* is a probiotic strain that produces several extracellular enzymes to augment digestibility and absorption of nutrients in addition to overall intestinal immune function (Gould et al. 1975; Gracia et al. 2003; Lee et al. 2008). Due to its higher resistance to harsh environments, *Bacillus amyloliquefaciens* is preferred as feed supplement (Hong et al. 2005).

China is the largest antibiotics producer and consumer in the world and large amount of antibiotics were applied in livestock industries (Hvistendahl 2012). However, the use of antibiotics in feed is poorly monitored (Zhu et al. 2013). As the formal Ministry of Agriculture announcement (number 2428) regarding the cessation of colistin as a growth promoter (feed additive) in animal was released on July 26, more than 8000 tonnes of colistin as a growth promoter from the Chinese veterinary sector will be withdrew (Walsh and Wu 2016). Thus, it is urgent to find potential substitutes for antibiotics. A great number of reports demonstrated that probiotics perform better than antibiotics in pig industry. According to Choi et al. (2011), multimicrobe probiotic increased apparent total tract digestibility of gross energy in pigs compared to the aureomycin-fed ones. Wang et al. (2012a) also found that both *L. fermentum* I5007 and aureomycin can decrease apoptosis in pig gastrointestinal tract, but *L. fermentum* I5007 exhibited additional effects in alleviating weaning stress syndrome. However, others had some different results. Guerra et al. (2007) observed that the best growth performance results were obtained in pigs receiving antibiotic rather than probiotics. And probiotics can perform similarly to antibiotics in weaned pigs in high-health status farms (Kritas and Morrison 2005). It is well-known that piglets can encounter many stressors, including pathogens and mold-contaminated feed (Sugiharto et al. 2014; Yin et al. 2014, 2015), which may cause severe inflammatory reaction and unbalance the antioxidant system. It was thus of interest to determine if the replacement of antibiotics with probiotics can ameliorate the oxidative stress in piglets. Autophagy is considered to engage in the cross-talk with oxidative stress in both cell signaling and protein damage (Lee et al. 2012). Therefore, the objective of this study was to evaluate effects of probiotic *Bacillus amyloliquefaciens* as a substitute for antibiotics on growth performance, antioxidant ability and intestinal autophagy of piglets. The underlying molecular mechanisms will provide a theoretical basis for the usage of probiotics as antibiotic alternatives in pig industry in China.

## Materials and methods

### Animals and diets

Ninety male piglets (Duroc × Landrace × Yorkshire) (42 days old) with similar initial weights were randomly divided into three groups. Each group had three replicates with ten pigs per replicate. All pigs were fed ad libitum. The experiment was approved by and performed in accordance with the guidelines of the local ethics committee. The basal diet was supplemented with minerals and vitamins to meet or exceed the requirements for pigs (NRC 1998). Piglets in Group 1 (G1) were fed with the normal diet containing 150 mg/Kg aureomycin. Piglets in Group 2 (G2) were fed with the diet containing 75 mg/Kg aureomycin and 1 × 10^8^ cfu/Kg *Ba*, while piglets in Group 3 (G3) were fed with the diet containing 2 × 10^8^ cfu/Kg *Ba* without any antibiotics. The experimental period was 28 days. Initial and final body weights were recorded. The basal diet of piglets was prepared according to NRC 1998 and the composition and nutrient levels of the basal diets are listed in Table 1.

**Table 1:** Composition and nutrient levels of basal diet

Ingredients	Contents (%)	Nutrition levels	Contents (%)
Corn	61.25	CP	19.00
Soybean meal	15.79	DE/(MJ/Kg)	14.11
Extruded-soybean	10.00	Calcium	0.80
Imported fish meal	5.00	TP	0.63
Wheat bran	3.00	AP	0.40
Soybean oil	1.74	Lysine	1.15
Premix	1.00	Methionie + cysteine	0.67
Limestone	0.98	Threonine	0.77
CaHPO_4_	0.78	Tryptophan	0.22
Salt	0.37		
Lysine-HCl	0.09		
Total	100.00		

Providing the following amount of vitamins and minerals per kilogram on an as-fed basis: Zn (ZnO), 50 mg; Cu (CuSO_4_), 20 mg; Mn (MnO), 55 mg; Fe (FeSO_4_), 100 mg; I (KI), 1 mg; Co (CoSO_4_), 2 mg; Se (Na_2_SeO_3_), 0.3 mg; vitamin A, 8255 IU; vitamin D_3_, 2000 IU; vitamin E, 40 IU; vitamin B_1_, 2 mg; vitamin B_2_, 4 mg; pantothenic acid, 15 mg; vitamin B_6_, 10 mg; vitamin B_12_, 0.05 mg; vitamin PP, 30 mg; folic acid, 2 mg; vitamin K_3_, 1.5 mg; biptin, 0.2 mg; choline chloride, 800 mg; and vitamin C, 100 mg

*CP* crude protein, *De* digestible energy, *TP* total phosphorus, *AP* available phosphorus

### Bacterial strain and aureomycin

*Bacillus amyloliquefaciens* cells (China Center For Type Culture Collection No: M 2012280) (1 × 10^8^ cfu/g) were prepared by the Laboratory of Microbiology, Institute of Feed Sciences, Zhejiang University, China. Starch was used to dilute *Ba* and the same amount of starch was also added to each group to compensate for the difference in nutrient composition of the diets. Aureomycin was obtained from Tongyi feed agriculture and animal husbandry Co., Ltd. (Qingdao, China).

### Sample collection

At the end of the experiment, piglets (n = 6) were randomly picked from each group to collect the samples. After 12 h fasting, blood samples were collected from the vena cava anterior and were centrifuged for 10 min at 4 °C (3000×*g*, Centrifuge 5804R, Eppendorf, Germany). Mid-jejunal segments were carefully dissected and rinsed with sterilized saline. Jejunal mucosa samples were gently scraped off. All samples were placed in liquid nitrogen immediately and then stored at −70 °C for further analysis.

### Western blotting

Extracted intestine proteins were separated by electrophoresis (Bio-Rad) on SDS-PAGE before being transferred electrophoretically to a nitrocellulose membranes membrane. After blocking with no protein blocking solution (Sangon Biotech), the membranes were incubated with a primary antibody overnight at 4 °C. After washing with TBST, membranes were incubated with secondary antibody linked to HRP. The blots were then developed with an ECL detection system as per the manufacturer’s instructions. Rabbit anti-Nrf2 and anti-p47^*phox*^ polyclonal antibodies was purchased from Santa Cruz Biotechnology (CA, USA). Rabbit anti-Nrf2 (phosphor S40) and anti-Akt monoclonal antibodies as well as anti-mTOR polyclonal antibody were obtained from Abcam (MA, USA). Rabbit anti-Keap1, anti-p62, anti-Akt (phosphor S473) monoclonal antibodies as well as anti-mTOR (phosphor S2448) polyclonal antibody were purchased from Cell Signaling Technology (MA, USA). Rabbit anti-LC3 monoclonal antibody was obtained from Sigma (MO, USA). Mouse anti-β-actin monoclonal antibody was obtained from Biotime Biotechnology (China). The IgG-HRP secondary antibodies were purchased from Biotime Biotechnology (China).

### Biochemical analyses

Jejunal mucosa samples were homogenized with ice-cold physiologic saline (1:10, w/v) and centrifuged at 2000*g* for 10 min. Supernatants were collected for determination of the total anti-oxidant capability (T-AOC), concentrations of glutathione (GSH) and malondialdehyde (MDA) and the activities of superoxide dismutase (SOD), glutathione peroxidase (GSH-Px) and nicotinamide adenine dinucleotide phosphate oxidase (NOX), using kits purchased from Nanjing Jiancheng Bioengineering Institute (Nanjing, China). Enzyme-linked immunosorbent assay (ELISA) kits for 8-hydroxy-2′-deoxyguanosine (8-OHdG) was purchased from Bioleaf Biological Co., Ltd. (Shanghai, China). All the above parameters were determined by spectrophotometry according to the manufacturers’ instructions (Lei et al. 2015).

### RNA extraction and real-time quantitative PCR

Total RNA isolated from intestine (RNAiso plus, TAKARA) was reverse-transcribed using PrimeScript II 1st Strand cDNA Synthesis Kit (TAKARA). Real-time PCR was performed using SYBR Premix Ex Taq II (TAKARA) and the ABI 7500 real-time PCR system (Applied Biosystems). The thermocycle protocol lasted for 30 s at 95 °C, followed by 40 cycles of 5-s denaturation at 95 °C, 34-s annealing/extension at 60 °C, and then a final melting curve analysis to monitor purity of the PCR product. Primer sequences were designed and selected by Primer 5.0 and Oligo 7.0 softwares and the sequences are presented in Additional file 1: Table S1. The 2^−∆∆Ct^ method was used to estimate mRNA abundance. Relative gene expression levels were normalized to those of the eukaryotic reference gene *GAPDH*.

### Statistical analysis

Data are presented as means with their standard deviation. They were analyzed with SPSS 16.0 for Windows, using ANOVA, Tukey’s test. Differences were considered statistically significant at *p* < 0.05 or 0.01.

## Results

### Replacing antibiotics with *Ba* improved pig growth performance

As shown in Table 2, piglets in G2 and G3 had higher average daily gain compared to that of G1 (628.57 ± 19.88 vs 555.71 ± 14.71 and 613.32 ± 13.36 vs 555.71 ± 14.71, respectively). The daily feed intake was also elevated in piglets receiving probiotics, but there was no significant difference for the final body weight among three groups.

**Table 2:** Effect of *Ba* on growth performance of piglets (n = 3 replicates)

Items	G1	G2	G3
Initial body weight (kg)	14.62 ± 0.203	14.20 ± 0.18	14.89 ± 0.38
Final body weight (kg)	30.18 ± 1.67	31.80 ± 0.53	32.07 ± 0.86
Daily feed intake (g)	902.48 ± 20.35^b^	1022.48 ± 22.44^a^	942.69 ± 27.78^a^
Average daily gain (g)	555.71 ± 14.71^b^	628.57 ± 19.88^a^	613.32 ± 13.36^a^
Feed: gain	1.624 ± 0.036	1.627 ± 0.035	1.537 ± 0.067

Data are expressed as mean ± SD (n = 3 replicates). Different letters indicate a statistically significant difference between groups (*p* < 0.05)

### Antioxidant profiles in serum of piglets

Compared to G1, we observed that replacing half antibiotics with *Ba* (G2) significantly elevated the serum T-AOC, which was paralleled by the increased GSH level, SOD and GSH-Px activities. Similarly, higher T-AOC in G3 was also found, which was accompanied by improved GSH level, SOD and GSH-Px activities. Further, GSH levels in G3 were much higher than that of G2. 8-OHdG levels were markedly decreased in G3 compared to control piglets (Table 3).

**Table 3:** Effects of *Ba* on serum and jejunum antioxidant parameters (n = 6)

Parameters	G1	G2	G3
Serum			
T-AOC (U/mL)	7.00 ± 0.81^b^	8.68 ± 0.58^a^	8.52 ± 1.36^a^
GSH (mg/L)	1.88 ± 0.08^c^	2.60 ± 0.04^b^	3.77 ± 0.10^a^
SOD (U/mL)	55.49 ± 1.50^b^	79.07 ± 3.12^a^	71.15 ± 1.14^a^
GSH-Px (U/mL)	692.06 ± 32.95^b^	854.58 ± 65.51^a^	859.6 ± 49.21^a^
8-OHdG (ng/mL)	29.1 ± 6.42^a^	21.1 ± 0.93^a^	12.57 ± 6.95^b^
MDA (nmol/ml)	23.91 ± 3.57	23.17 ± 0.57	23.04 ± 0.13
Jejunum			
T-AOC (U/mL)	0.14 ± 0.02^b^	0.25 ± 0.14^b^	0.79 ± 0.09^a^
GSH (mg/L)	4.08 ± 1.26^ab^	4.88 ± 1.38^a^	3.21 ± 0.51^b^
SOD (U/mL)	23.95 ± 1.57	24.42 ± 2.32	23.57 ± 1.46
GSH-Px (U/mL)	92.94 ± 16.09^b^	44.22 ± 11.35^c^	119.93 ± 9.25^a^
8-OHdG (ng/mL)	1.55 ± 0.22^a^	1.39 ± 0.09^b^	2.10 ± 0.73^a^
MDA (nmol/ml)	0.64 ± 0.10^a^	0.44 ± 0.22^b^	0.35 ± 0.13^b^

Data are expressed as mean ± SD (n = 6). Different letters indicate a statistically significant difference between groups (*p* < 0.05)

### Antioxidant profile and expression of genes related to antioxidation in jejunal mucosa of piglets

**Fig. 1:**
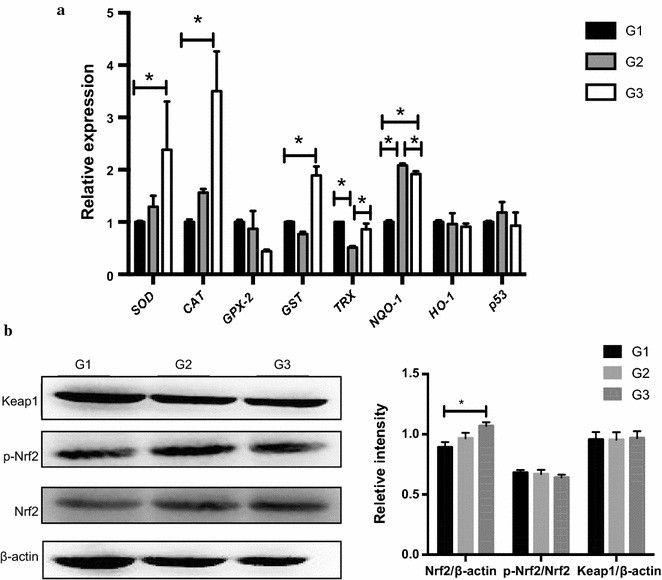
Effects of *Ba* on antioxidant gene expressions (**a**) and Nrf2/Keap1 signaling pathway (**b**) in jejunum (n = 3). Gene expressions of *SOD*, *CAT, GPX*-*2, GST*, *TRX*, *NQO*-*1*, *HO*-*1* and *p53* were detected by real time PCR. Total protein levels of Keap1 and β-actin as well as the phosphorylated and total protein levels of Nrf2 in the jejunum of piglets were determined using Abs recognizing phospho-specific or total protein. Results are given as mean ± SD. Differences between groups were determined by one-way ANOVA followed by Tukey test. Mean values were significantly different: *p < 0.05

Compared to G1, T-AOC in the jejunal mucosa of G2 piglets was slightly increased. Meanwhile, GSH-Px activity, 8-OHdG level and MDA concentration were markedly reduced. T-AOC in G3 was dramatically increased owing to the improved GSH-Px activity. Although 8-OHdG levels in G3 were not altered, the MDA content was significantly decreased (Table 3). RT-qPCR results of the antioxidant genes in jejunal mucosa showed that compared to G1, the thioredoxin reductase (*TRX*) gene expression in G2 was markedly down-regulated, while NAD(P)H: quinone oxidoreductase 1 (*NQO*-*1*) transcription was up-regulated. Moreover, gene expressions of *SOD*, catalase (*CAT*), glutathione-S-transferase (*GST*) and *NQO*-*1* in G3 were increased significantly. *NQO*-*1* transcript level in G3 was much lower than that of G2, whereas *TRX* was much higher (Fig. 1).

### Replacing antibiotics with *Ba* activated Nrf2/Keap1 signaling pathway in jejunal mucosa of piglets

Glutathione synthesis and antioxidant enzymes, such as CAT, SOD, HO-1 and GSH-Px, can be regulated via Nrf2/kelch-like ECH-associated protein 1 (Keap1) signaling pathway (Itoh et al. 1997; Cho et al. 2006; Riedl et al. 2009). It was found that Nrf2 level was significantly improved in G3 compared to G1, although there was no significant difference among three groups in the Nrf2 phosphorylation and Keap1 expression (Fig. 1).

### Effects of replacing antibiotics with *Ba* on MAPKs signaling pathways

**Fig. 2:**
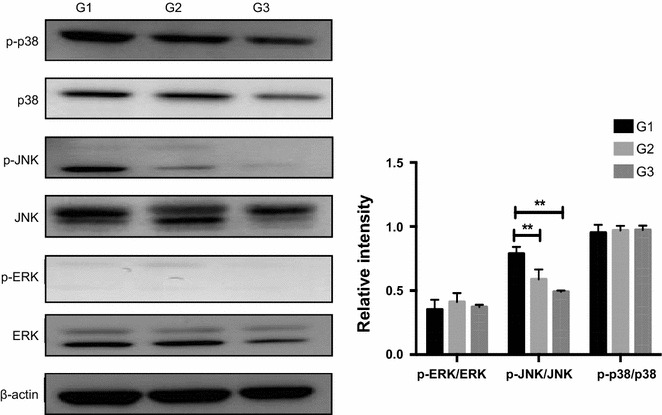
Effects of *Ba* on MAPK signaling pathways in the jejunum of piglets. Phosphorylated and total protein levels of p38, JNK, ERK and β-actin in the jejunum of piglets were determined using Abs recognizing total protein. Results are given as mean ± SD. Differences between groups were determined by one-way ANOVA followed by Tukey test (n = 3). Mean values were significantly different: **p < 0.01

Mitogen-activated protein kinases (MAPKs) are integral part of the response to a variety of stresses (Inoue et al. 2005; Dhingra et al. 2007). Here, the extracellular signal-regulated kinases 1/2 (ERK1/2) and p38 MAPK were not activated in *Ba*-fed piglets as well, whereas replacing antibiotics with *Ba* in G2 and G3 markedly down-regulated the phosphorylation level of JNK (Fig. 2), implying the inhibition of JNK signaling pathway.

### NOX activity and expression in jejunal mucosa of piglets

**Fig. 3:**
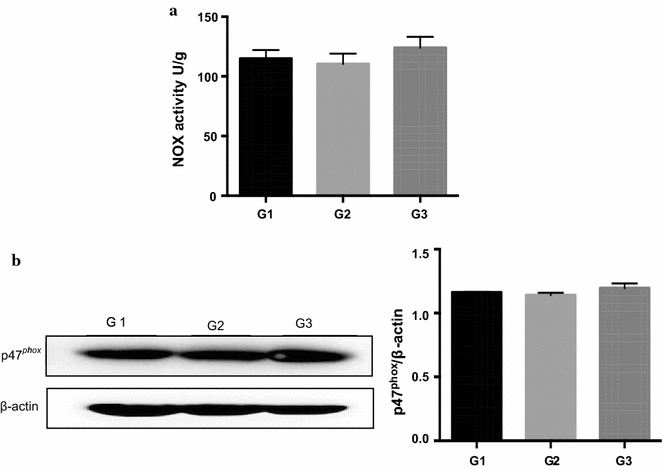
Effects of *Ba* on NOX activity and expression in the jejunum of piglets. **a** NOX activity, **b** p47^*phox*^ expression. Total protein levels of p47^*phox*^ and β-actin in the jejunum of piglets were determined using Abs recognizing total protein. Results are given as mean ± SD. Differences between groups were determined by one-way ANOVA followed by Tukey test (n = 3)

As shown in Fig. 3, no significant difference of NOX activity was found when antibiotic was replaced by *Ba*. Similarly, the expression of p47^*phox*^, an active subunit of NOX, which plays an important role in ROS production, also remained unchanged.

### Replacing antibiotics with* Ba* induced autophagy in jejunal mucosa of piglets

**Fig. 4:**
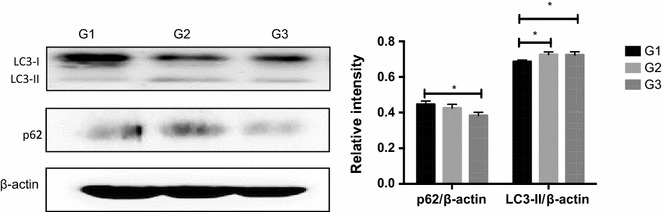
Effects of *Ba* on autophagy in the jejunum of piglets. Total protein levels of LC3, p62 and β-actin in the jejunum of piglets were determined using Abs recognizing total protein. Results are given as mean ± SD. Differences between groups were determined by one-way ANOVA followed by Tukey test (n = 3). Mean values were significantly different: *p < 0.05

In mammals, LC3 has been widely used as a sole marker of autophagy, and p62 degradation correlates with autophagic flux (Kabeya et al. 2000; Mizushima et al. 2010). In the present study, replacing antibiotics with *Ba* in G2 and G3 induced higher LC3-II/β-actin expression. Furthermore, p62 expression was markedly decreased in G3 (Fig. 4).

### Effects of replacing antibiotics with* Ba* on PI3K/Akt/mTOR signaling pathways

**Fig. 5:**
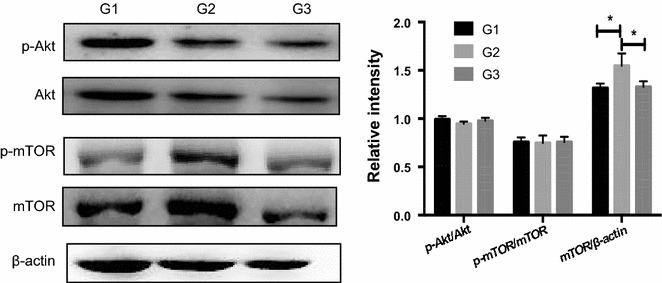
Effects of *Ba* on Akt/mTOR in the jejunum of piglets. Phosphorylated and total protein levels of Akt, mTOR and β-actin in the jejunum of piglets were determined using Abs recognizing total protein. Results are given as mean ± SD. Differences between groups were determined by one-way ANOVA followed by Tukey test (n = 3). Mean values were significantly different: *p < 0.05

Phosphatidylinositol 3-kinase (PI3K)/protein kinase B (Akt)/mammalian target of rapamycin (mTOR) signaling pathway has been proved to regulate the formation of autophagosome (Sui et al. 2014). In Fig. 5, there were no significant differences in activation of Akt and mTOR among three groups, but piglets in G2 showed a higher mTOR expression in jejunum.

## Discussion

Problems such as antibiotic resistance and antibiotic residues caused by the abuse of antibiotics have been frequently reported worldwide. As green feed additives (Chen et al. 2013), probiotics have been widely promoted as alternatives to replace in-feed antibiotics due to their abilities to improve livestock production, efficiency and welfare (Bocourt et al. 2004; Dersjant-Li et al. 2013). However, the impact of probiotics on the antioxidant system of piglets remains unclear. Thus, we evaluated the effects of probiotic *Bacillus* as a substitute for antibiotics on antioxidant capacity of piglets.

In the present study, the daily weight gain of piglets in *Ba*-fed groups was significantly improved compared to the antibiotic group. The major antioxidant defense machineries are composed of antioxidant enzymes and biological antioxidants (Itoh et al. 1997; Cho et al. 2006; Riedl et al. 2009). Our results revealed that the serum T-AOC and SOD activities and GSH levels were significantly enhanced in *Ba*-fed groups, while 8-OHdG concentrations were markedly decreased in piglets receiving only *Ba* without any antibiotic. Intestinal epithelial redox environment is central to the functions of the organ in nutrient digestion and absorption (Circu and Aw 2012), so the redox status of intestine is of vital importance for animal health. According to the antioxidant profiles in jejunal mucosa, replacing all antibiotics with *Ba* in G3 significantly increased T-AOC due to the increase of GSH-Px activity, contributing to lowered MDA concentrations. These results were in agreement with other findings (Wang et al. 2009; Yang et al. 2009; Wang et al. 2012b; Tang et al. 2016), which showed that the antioxidase activities were enhanced while MDA levels were decreased by probiotics supplementation. To gain a clear depiction of antioxidant status, we also measured the antioxidant gene expressions in jejunum. Replacing all antibiotics with *Ba* induced higher *SOD*, *CAT*, *GST*, *NQO*-*1* mRNA levels, however, piglets in G2 (replacing half antibiotics with *Ba*) showed lowered *TRX* transcription. Given that TRX is involved in DNA and protein repair (Lu and Holmgren 2014), it can be deduced that the down-regulated *TRX* expression in this study indicated less DNA and protein damage.

The Nrf2–Keap1 signaling pathway is one of the most important cell defense and survival pathways. Nrf2 is primarily regulated by Keap1, a substrate adaptor for a Cul3-containing E3 ubiquitin ligase. Oxidative stress or antioxidants can cause a conformational change in Keap1-Cul3-E3 ubiquitin ligase by acting on specific cysteine residues in Keap1 (Zhang 2006). This change can stabilize Nrf2 and promote the free Nrf2 to translocate into nucleus, where it binds to a DNA promoter and initiates transcription of many detoxifying and antioxidant genes (Jaramillo and Zhang 2013; Jones et al. 2015). In the present study, replacing all antibiotics with *Ba* significantly up-regulated Nrf2 expression. It is known that antioxidant genes, including *SOD*, *CAT*, *GST* and *NQO*-*1*, are Nrf2 target genes. As aforementioned, consistent with the Nrf2 expressions, transcript levels of these genes were also elevated by *Ba* administration. Similar to our results, previous research also showed that Nrf2-Keap1 signaling pathway could be activated by probiotics to ameliorate the oxidative damage in epithelial of Drosophila, HT-29 cells and obese mice (Gao et al. 2013; Chauhan et al. 2014; Jones et al. 2015). Although it is generally accepted that modification of the Keap1 critical cysteine residues is a chemico-biological trigger for the activation of Nrf2, some literature has revealed alternative mechanisms of Nrf2 regulation, including phosphorylation of Nrf2 (Bryan et al. 2013). However, here we did not observe significant differences in p-Nrf2 levels among three groups. Thus, according to the commentary of Bryan et al. (2013), we speculate that *Ba* activated Nrf2 in a Keap1-dependent way by altering Keap1 conformation.

MAPKs, including p38 MAPK, JNK, and ERK1/2, have also been shown to influence a wide range of cellular responses (Shifflett et al. 2004) via regulating transcription factors, such as AP-1, NFκB and FoxOs (Sui et al. 2014). In this study, no obvious changes were found in p38 MAPK and ERK1/2 expressions while JNK was decreased by *Ba* treatment compared with antibiotics. JNK is an evolutionarily conserved signal transduction system that can be triggered by several types of external insults, including oxidative stress (Davis 2000; Weston and Davis 2007; Barr and Bogoyevitch 2011). Evidence demonstrated that antioxidants could inhibit JNK activation in rats aortic smooth muscle cells (Kyaw et al. 2001) and remote noninfarcted myocardium (Li et al. 2001). Increased JNK activity in the obese mice was also abolished during probiotic administration (Toral et al. 2014). Therefore, the decreased JNK expression may be linked to the lowered level of oxidative stress induced by *Ba* addition.

Oxidative stress is derived either from an increase in ROS production or decreased levels of ROS-scavenging proteins. Therefore, the activity of NOX, a multi-subunit protein complex that regulates the transfer of electrons across biological membranes to generate downstream ROS (Bedard and Krause 2007) was measured. Among all the NOX subunits, the cytosolic subunit p47^*phox*^ is necessary for NOX activation and regulation (Clark et al. 1990; Quinn et al. 1993; El-Benna et al. 1994). Rashid et al. (2014) suggested that probiotics VSL#3 protected rats from endothelial dysfunction in rats by down-regulating p47^*phox*^ expression. Tapia-Paniagua et al. (2015) also reported that probiotic SpPdp11 decreased the *NOX* transcription in *Solea senegalensis*. However, in this study, *Ba* replacement didn’t alter NOX activity and p47^*phox*^ level in piglets. Collectively, replacement of antibiotics with *Ba* could improve antioxidant status in serum and jejunum of piglets via activating Nrf2 signaling pathway and, in turn, the activities and gene expressions of antioxidases were increased. This effect was more obvious in group replaced all antibiotics with *Ba*.

Under certain stress, defensive mechanisms are often not enough to completely avoid cellular injury, and autophagy, a second line of defense, is required for the repair and removal of damaged components (Navarro-Yepes et al. 2014). When autophagy is activated, LC3 is cleaved to proteolytic derived LC3-II (Gonzalez-Polo et al. 2007). p62, an autophagy adaptor protein, can bind to LC3-II to facilitate degradation of ubiquitinated protein aggregates in autolysosomes (Kang et al. 2011). Thus, detection of LC3-II and p62 can be used to estimate the induction of autophagy. Results from this study revealed that LC3-II expressions were obviously enhanced while p62 level was significantly reduced following *Ba* replacement, suggesting an increase in autophagic activity. Although autophagy is a process that cells response to stress or stimuli, it is involved in both cell death and cell survival depending on the cell type and strength of specific stimuli (Janku et al. 2011). Research indicated that antioxidants may exert the protective role by increasing autophagy level. Resveratrol, a natural polyphenolic compound with potent antioxidant properties (Baur and Sinclair 2006), has been shown to promote longevity through the Sirtuin-1-dependent induction of autophagy (Morselli et al. 2010). tBHQ, a well-known antioxidant, can protect hepatocytes against lipotoxicity via inducing autophagy (Li et al. 2014). In the opinion of Morselli et al. (2010), as a possibility, increased autophagy might improve cellular resistance to stress by augmenting the metabolic buffering capacity of cells. Thus, the probiotic *Ba*, as a mild activator, may increase autophagy level to elevate the resistance to oxidative stimuli.

The classical pathway that regulates autophagy involves the serine/threonine kinase (AKT), mammalian target of rapamycin (mTOR). PI3K-Akt transduction serves as a critical signaling axis in cell growth, proliferation, and cell survival (Tsai et al. 2015). mTOR is the major downstream target of Akt and the inhibition of PI3 K-Akt-mTOR signaling pathway plays important roles to activate autophagy (Pattingre et al. 2008; Zhang et al. 2016; Pang et al. 2016). In our experiments, the phosphorylation levels of Akt and mTOR were not regulated by *Ba* replacement significantly, but mTOR expression was significantly enhanced in G2. Although autophagy is negatively regulated by mTOR, several pathways seem to regulate autophagy in mammalian cells. Autophagy can be induced by lowering intracellular inositol or inositol 1,4,5-trisphosphate (IP_3_) levels, which was the first demonstration of the existence of an autophagy pathway in mammalian system independent of mTOR (Sarkar et al. 2005). According to the review of Sarkar et al. (2009), many autophagy enhancers, like loperamide, verapamil, 2′5′-dideoxyadenosine, trehalose, small molecule enhancer of rapamycin 10, can exert their protective effect in a mTOR-independent way. Similar to our results, in the recent study of Zhou et al. (2016), sulforaphane treatment inhibited rotenone-induced oxidative stress, increased Nrf2 expression, attenuated rotenone-inhibited mTOR-mediated signaling pathway and rescued rotenone-inhibited autophagy. In their views, the interplay between mTOR and autophagy is complex. Although changes in mTOR signaling are related to autophagy, the relationship between sulforaphane, mTOR signaling, and autophagy processes does not seem mutually dependent. Thus, we speculate that in the present study, *Ba* elevated the autophagy level in a mTOR-independent manner. Our results also demonstrated that *Ba* effectively increased Nrf2 level, leading to the enhancement of antioxidant gene expressions. In recent years, a growing body of evidence has suggested that Nrf2 is related to mTOR. Zhou et al. (2016) revealed that sulforaphane exerted neuronal protective effects via activating Nrf2 and mTOR. Zhang et al. (2014) found that salvianolic acid A-mediated Nrf2 activation was dependent on the activation of mTORC1. So, we hypothesize that the oxidative stress of piglets receiving *Ba* as aureomycin substitute was ameliorated via activation of Nrf2 and mTOR. Taken together, the enhanced mTOR level induced by *Ba* might be considered as a mechanism to resist oxidative stress rather than regulating autophagy.

In conclusion, these findings highlighted the crucial role of *Ba* in enhancing the antioxidant capacity of piglets via activating Nrf2 signaling pathway and intestinal autophagy. Although the control group without antibiotics and *Ba* was absent in our study, negative control was also not included in some researches evaluating the effects of probiotics as antibiotic substitutes (Kritas and Morrison 2005; Silva et al. 2010). Besides, in-feed antibiotics have been proved to contribute to a 3–5% improvement in nutrient utilization, a 3–8% improvement in growth rate, and a 2–5% improvement in feed conversion efficiency (Close 2000). When compared to antibiotics, *Ba* benefited superior to antibiotics in the current study. So it could be said that the *Ba* used here could be a feasible alternative to antibiotic, with the capacity of improving pig performance and maintaining redox balance.

## Additional file

**Additional file 1: Table S1.** Gene name, primer sequences (F: forward, R: reverse) and product sizes.

## Abbreviations

*Ba*: *Bacillus amyloliquefaciens*
Nrf2: nuclear factor erythroid 2 related factor 2
T-AOC: total anti-oxidant capability
GSH: glutathione
MDA: malondialdehyde
SOD: superoxide dismutase
GSH-Px: glutathione peroxidase
NOX: nicotinamide adenine dinucleotide phosphate oxidase
8-OHdG: 8-hydroxy-2′-deoxyguanosine
ELISA: enzyme-linked immunosorbent assay
GAPDH: glyceraldehyde-3-phosphate dehydrogenase
GPX: glutathione peroxidase
CAT: catalase
GST: glutathione-S-transferase
TRX: thioredoxin reductase
HO-1: heme oxygenase 1
NQO-1: NAD(P)H: quinone oxidoreductase 1
LC3: microtubule-associated protein 1 light chain 3
JNK: c-Jun N-terminal kinase
ERK1/2: extracellular signal-regulated kinases ½
MAPK: mitogen-activated protein kinases
Keap1: kelch-like ECH-associated protein 1
PI3K: phosphatidylinositol 3-kinase
Akt: protein kinase B
mTOR: mammalian target of rapamycin
