# Supplementary material for: Effects of probiotic Bacillus as a substitute for antibiotics on antioxidant capacity and intestinal autophagy of piglets
Source: AMB Express. 2017 Feb 28;7:52. doi: 10.1186/s13568-017-0353-x (PMC5328899; doi:10.1186/s13568-017-0353-x)
Supplement: Supplementary file 1 — Additional file 1: Table S1. Gene name, primer sequences (F: forward, R: reverse) and product sizes. [file 13568_2017_353_MOESM1_ESM.docx]

**Supplementary materials**

**AMB express**

**Effects of probiotic *Bacillus* as a substitute for antibiotics on antioxidant capacity and intestinal autophagy of piglets**

^1^ Key Laboratory of Molecular Animal Nutrition of the Ministry of Education, Institute of Feed Science, College of Animal Sciences, Zhejiang University, Hangzhou 310058, China

^2^ Animal Nutrition and Human Health Laboratory, School of Life Sciences, Hunan Normal University, Changsha 410006, China

^*^ Corresponding author:

Weifen Li; Yali Li

Telephone: 86-571-88982108; Fax: 86-571-86091820; E-mail: [wfli@zju.edu.cn](mailto:wfli@zju.edu.cn); liyali06@163.com

**Table S1 Gene name, primer sequences (F: forward, R: reverse) and product sizes**

| Gene symbol | Gene name | GeneBank accession No. | Primer sequence | Product size (bp) |
| --- | --- | --- | --- | --- |
| *GAPDH* | glyceraldehyde-3-phosphate dehydrogenase | NM_001206359.1 | F:5'ATGGTGAAGGTCGGAGTGAAC3'  R:5'CTCGCTCCTGGAAGATGGG3' | 235 |
| *SOD-1* | superoxide dismutase 1 | NC_010455.4 | F: 5’GCAGGTCCTCACTTCAATCC3’  R: 5’CTTCCAGCATTTCCCGTCTG3’ | 248 |
| *GPX-2* | glutathione peroxidase 2 | NM_001115136.1 | F: 5’GGCAGTGCTGATTGAGAATGG3’  R: 5’CAGGTAGGCGAAGACAGGAG3’ | 271 |
| *CAT* | catalase | D89812.1 | F: 5’CACACATACCCATTCGTCACG3’  R: 5’CAGCCCTAACCTTCACTTACC3’ | 157 |
| *GST* | glutathione-S-transferase | AB000884.1 | F: 5’CAACCCAGAAGACTGCTCAAG3’  R: 5’GGACCACTCAAGGAATACAGAAG3’ | 159 |
| *TRX-1* | thioredoxin reductase 1 | NC_010443.4 | F: 5’CTTTACCTTATTGCCCGGGG3’  R: 5’GTTCACCGATTTTGTTGGCC3’ | 162 |
| *HO-1* | heme oxygenase 1 | NM_001004027.1 | F: 5’AGCTGTTTCTGAGCCTCCAA3’  R: 5’CAAGACGGAAACACGAGACA3’ | 130 |
| *p53* | p53 | NM_213824 | F: 5’CTGCTTCCTGAAAACAACC3’  R: 5’AAGGGACAAAGGACGACA3’ | 199 |
| *NQO1* | NAD(P)H: quinone oxidoreductase 1 | NM_001159613.1 | F: 5’CCAGCAGCCCGGCCAATCTG3’  R: 5’AGGTCCGACACGGCGACCTC3’ | 160 |
